# Epithelial Biological Response to Machined Titanium vs. PVD Zirconium-Coated Titanium: An In Vitro Study

**DOI:** 10.3390/ma15207250

**Published:** 2022-10-17

**Authors:** Lucia Memè, Davide Sartini, Valentina Pozzi, Monica Emanuelli, Enrico M. Strappa, Paolo Bittarello, Fabrizio Bambini, Gianni Gallusi

**Affiliations:** 1 Department of Clinical Sciences and Stomatology, Polytechnic University of Marche, 60126 Ancona, Italy; 2 Department of Clinical Sciences, Polytechnic University of Marche, 60126 Ancona, Italy; 3 Department of Health Technologies, IRCCS Ospedale Galeazzi-Sant’Ambrogio, University of Milan, 20157 Milan, Italy; 4 Independent Researcher, 06062 Città della Pieve, Italy; 5 Department of Clinical Sciences and Translational Medicine, University Tor Vergata, 00133 Rome, Italy

**Keywords:** epithelial attachment, zirconia coating, physical vapor deposition, abutment

## Abstract

The aim of this study was to compare the epithelial biological response to machined titanium Ti-6Al-4V grade 5 and titanium Ti-6Al-4V grade 5 coated with zirconia (ZrN) by physical vapor deposition (PVD). Human keratinocytes were cultured in six-well plates. Machined titanium TiAl4V4 grade 5 (T1) and ZrN-coated titanium TiAl4V4 grade 5 (T2) discs were placed in two different wells. The remaining two wells served as control (C). Scanning electron microscopy (SEM) and energy-dispersive spectroscopy (EDS) were performed to compare the T1 and T2 surfaces. Subsequent analyses were performed to explore the effect of T1 and T2 contact with human keratinocyte HUKE cell lines. Cell viability was evaluated using a trypan blue exclusion test and MTT assay. Cell lysates from C, T1, and T2 were Western blotted to evaluate E-cadherin and Integrin-α6β4 expression. SEM revealed that T2 was smoother and more homogeneous than T1. EDS showed homogeneous and uniform distribution of ZrN coating on T2. Cell viability analyses did not show significant differences between T1 and T2. Furthermore, E-cadherin and Integrin-α6β4 expressions of the epithelial cells cultured in T1 and T2 were similar. Therefore, titanium Ti-6Al-4V grade 5 surfaces coated with ZrN by PVD seem to be similar substrates to the uncoated surfaces for keratinocyte adhesion and proliferation.

## 1. Introduction

Osseointegrated implants have achieved a high level of predictability over both the medium and long term [1,2,3,4,5,6]. One of the keys to success is the formation and maintenance of a mucosal seal around dental implants [7,8,9]. The function of the soft tissues is to protect the underlying osseointegration from microbial challenge [10,11]. If the mucosal seal is compromised, osseointegration can be jeopardize [12].

Histological studies have shown differences between the morphology of the soft tissues around natural teeth and implants [13].

The epithelial and connective tissue attachment constitute the biological width [14]. The study of Tomasi et al. [15] on a human model reported that the epithelium extends 1.9 mm, while connective tissue extends 1.7 mm after 8 weeks of healing. These results were comparable to those of Berglund et al. [16] on an animal model.

Collagen fibers run parallel to an implant surface and do not attach to it [17]. On the contrary, the collagen fibers in dental sites run in different projections and are attached to the root cement [18]. The vascular supply of peri-implant soft tissues is lower than in dental sites since it is guaranteed only by the supra-periosteal vessels due to the absence of the periodontal ligament [19]. Near the implant surface, the vascular network is of a lesser extent than around the teeth [20]. Furthermore, the presence of abundant collagen fibers and the low quantity of fibroblasts has led to the definition of this area as “scar tissue” [21].

The histological findings explain why the soft tissues around the implants are more susceptible to inflammation induced by the bacterial biofilm and the resulting bone loss [19]. Studies on animal models have shown that the progression from mucositis to peri-implantitis is faster than that from gingivitis to periodontitis. Furthermore, destruction related to peri-implantitis is greater than that of periodontitis [22]. A recent meta-analysis has shown that the prevalence of peri-implant disorders related to inflammation is high and increasing [23].

Over the years, many efforts have tried to improve the quality of the mucous seal around implants [24] and have tested new biomaterials [25,26,27,28,29,30,31]. The studies concern both surgical procedures aimed at preventing or treating soft tissue complications and solutions related to abutment materials. Some authors have suggested the use of micro-grooves on the surface to implement cell adhesion [32], while others have coated the surface of the abutment with bioactive peptides [33]. Recently, coating titanium surfaces with zirconium nitride (ZrN) has gained interest from the scientific community. Several studies have shown that ZrN surface coating has antibacterial properties [34]. Consequently, an attempt was made to investigate whether zirconium coating was effective in influencing cellular viability [35].

The aims of the study were: (i) to compare the surface characteristics of titanium Ti-6Al-4V grade 5 coated with zirconia (ZrN) by physical vapor deposition (PVD) discs and machined titanium Ti-6Al-4V grade 5 uncoated discs by SEM and EDS; (ii) to compare the biological reaction of epithelial cells in terms of adhesion and proliferation to the two surfaces by cell viability tests (MTT and trypan blue analysis) and Western blot. The hypothesis was that surface treatment could improve epithelial cells adherence and proliferation.

## 2. Materials and Methods

### 2.1. Physical Vapor Deposition (PVD)

The present study was conducted using machined titanium Ti-6Al-4V grade 5 and titanium Ti-6Al-4V grade 5 discs coated with ZrN by PVD supplied by BioSAFin S.r.l. (Ancona, Italy). The discs were 8 mm in diameter and 3 mm thick.

PVD deposition of ZrN was carried out by Vacuum Surtec S.r.l. (San Giovanni in Croce, Cremona, Italy) using arc evaporation. The main parameters of the process were:

I. Temperature 80 °C;
II. Pressure of preparation to the process 2.5 × 10^−3^;
III. Pressure of deposition 2.5 × 10^1^;
IV. Time of deposition 180 s;
V. Thickness of the film 70 µm.

### 2.2. Scanning Electron Microscopy (SEM) and Energy-Dispersive Spectroscopy (EDS)

Scanning electron microscopy (SEM) was carried out by Department of Materials, Environmental Science and Urban Planning (Polytechnic University of Marche, Ancona, Italy). The analysis was performed using an SEM Philips XL-20 (FEI Italia SRL, Milan, Italy). The aim was to evaluate surface changes on the disks. Since electron microscopy is based on electron beams and the samples are conductive, it was not necessary to prepare the samples to increase their conductivity. The surfaces of the discs were analyzed at various magnifications: 150×, 1000×, and 3000×.

Energy-dispersive spectroscopy (EDS) allows us to monitor the elements present on the sample surface by studying the characteristic X-rays. The analysis was performed using an SEM Philips XL-20 (FEI Italia SRL, Milan, Italy) to get more information regarding the surface of the samples. The data obtained were processed using the “PHOENIX” software. Through this process, a distribution map of the elements was elaborated in greyscale. Titanium (Ti) and zirconium (Zr) were presented in red and green, respectively. Each element was presented with different color intensity according to its distribution (element-rich area or element-deficient area).

### 2.3. Cell Cultures and Incubation with Discs

The machined titanium Ti-6Al-4V grade 5 discs (T1) and zirconium-coated titanium Ti-6Al-4V grade 5 discs (T2) were washed with 96% ethanol for 10 min and left under ultraviolet radiation for 15 min by using a UV-c lamp prior to contact with cells within the plate well.

HUKE human immortalized keratinocytes cells were grown in EpiGRO™ Human Epidermal Keratinocyte Complete Culture Medium (Merck, Darmstadt, Germany) in the presence of 10% FBS (Euroclone, Milan, Italy) and 50 μg/mL gentamicin (Euroclone, Milan, Italy) in an incubator at 37 °C in a humidified atmosphere with 95% O_2_ and 5% CO_2_. The cell lines used in the study come from INNOPROT (Spain). In particular, immortalized human keratocytes (IM-HK) were provided by Innoprot and were developed by immortalizing primary human keratocytes (also known as corneal fibroblasts) with the HPV16 E6/E7 gene (reference: P10872-IM).

The frozen HUKE cells were stored in liquid nitrogen until use. The vial containing the frozen cells (1 × 10^6^) was first thawed in a water bath at 37 °C and centrifuged at 1200 rpm for 6 min. The cell pellet was resuspended in 10 mL of culture medium and transferred to a T-25 cm^2^ cell culture flask coated with 0.67 μg/cm^2^ collagen IV (Corning, Glendale, Arizona, USA) until 70–80% confluence was achieved. The second cell expansion was carried out by seeding cells at a density of 5 × 10^3^ cells per cm^2^ until reaching a confluence of 70–80% to prevent the irreversible contact inhibition obtained in a complete cellular monolayer. At this point, cells were ready to be replicated and used for further experiments.

Upon reaching the desired confluence, for each of the above reported passages, the culture medium was removed from the flask, and the cells were washed with 5 mL of D-PBS (Dulbecco phosphate-buffered saline solution) without calcium and magnesium. After buffer removal, cells were incubated with 1 mL of pre-warmed 0.05% trypsin-EDTA (Euroclone, Milan, Italy) for 5–10 min at room temperature in order to detach cells, and 5 mL of growth medium with trypsin inhibitor was added to the suspension. The sample was then centrifuged for 5 min at 1300 rpm, and the cell pellet was resuspended in culture medium and transferred to a new flask.

Following the above procedure, HUKE cells were cultured in polyethylene 6-well plates (Euroclone, Milan, Italy) alone or in contact with T1 and T2. A total of 2 × 10^4^ cells were seeded in each well, for a total of 6 wells (each sample in duplicate). The next day, the machined titanium Ti-6Al-4V grade 5 discs were inserted into 2 wells (T1), while the zirconium-coated titanium Ti-6Al-4V grade 5 discs were inserted into the 2 adjacent wells (T2). The control consisted of only HUKE cells in the remaining 2 wells (C). Cells were incubated with discs for 72 h. Cells were put in contact with discs within a total of 12 six-well plates in order to have enough samples to perform the following tests: trypan blue test (3 six-well plates), MTT assay (3 six-well plates), and Western blot (3 six-well plates).

### 2.4. Cell Viability Analysis

Cell viability analysis was performed using tests with trypan blue [36] and MTT [37,38,39] in order to assess any toxicity of the materials tested.

Cell viability was evaluated at 24, 48, and 72 h by counting the number of cells in the monolayer using the trypan blue exclusion test. Trypan blue (Merck, Darmstadt, Germany) is a dye capable of selectively staining dead cells, since viable cells, having an intact membrane, do not allow this dye to penetrate the cytoplasm. On the contrary, the dye easily penetrates dead cells, making them distinguishable from living cells with microscopic observation. Within each plate well, cells in contact with discs were removed through trypsinization, diluted in 0.4% trypan blue solution, loaded into a Bürker chamber, and observed via light microscopy.

MTT assay (3-dimethylthiazol-2,5-diphenyltetrazolium bromide) is a colorimetric test to estimate the number of cells with mitochondrial activity and, consequently, the viability of the cells. This test is based on a metabolic indicator, the soluble salt tetrazolium (yellow), which is reduced in the mitochondrion of vital cells by active dehydrogenase enzymes to form a blue–purple crystal that is insoluble in water (formazan). The solubilized crystals are quantified with a colorimetric method at a wavelength of 570 nm (reduced dye absorbance) with background correction at 690 nm.

At different timepoints, the culture medium was removed, and 200 μL of a solution containing MTT (Merck, Darmstadt, Germany) (5 mg/mL in medium without phenol red) and 1.8 mL of medium were added to the monolayer cells. The plates were then incubated at 37 °C for 4 h.

After removing the supernatant, the blue–violet crystals of formazan were dissolved by adding 2 mL of solvent (4% HCl in isopropanol) and quantified with a spectrophotometer (Secoman, Anthelie light, version 3.8, Contardi, Italy) at 570 nm and 690 nm.

Each experiment was repeated three times with each pair of samples, resulting in 6 samples per test group for statistical evaluation.

### 2.5. Fluorescence (DAPI)

DAPI (4′,6-diamidino-2-phenylindole) colors cell nuclei fluorescent blue due to its high affinity with double-stranded DNA (dsDNA). The link between DAPI and dsDNA, especially at the level of the AT clusters, produces a fluorescence about twenty times more intense than that of the background. Its use in fluorescent microscopy allowed for the identification of significant differences in nuclei due to treatment with discs.

DAPI (Euroclone, Milan, Italy) was diluted in PBS up to a concentration of 300 nM. The cell culture medium was aspirated from the cultured cells, which were washed 3 times in PBS (phosphate-buffered saline) (Bio-Optica M107, Milan, Italy) for 5 min each time. The cells were then fixed for 10 min in 3.7% formaldehyde (Merck, Darmstadt, Germany). After aspirating the fixative, the cells were washed again in PBS 3 times. The cells were permeabilized by using 0.2% Triton X-100 (Merck, Darmstadt, Germany). After aspirating the Triton X-100, the cells were washed again in PBS 3 times. Subsequently, the cells were incubated with DAPI. A total of 300 μL of the solution was applied to each section to completely cover the tissue. After a 5 min incubation, the samples were washed for 20 min in PBS. Finally, the excess buffer was removed, and an assembly was performed with a coverslip.

Optical fluorescence microscopy with DAPI was performed by the Department of Materials, Environmental Science and Urban Planning (Polytechnic University of Marche, Ancona, Italy) using a Zeiss Axio Imager A2 Microscope. For the DAPI, we used an excitation wavelength of 358 nm and emission at 461 nm.

### 2.6. Western Blot

Cell lysates from pellets related to T1 and T2 samples were collected after 72 h of culture for Western blot. The proteins were extracted with T-PER Tissue-Protein Extraction Reagent (Pierce, Rockford, IL) containing protease inhibitors (Sigma Chemical Co, St. Louis, MO). Protein concentration was determined with a Bradford assay (Merck, Darmstadt, Germany) protein using BSA (Merck, Darmstadt, Germany) as the standard. A total of 30 μg of proteins were solubilized in a Laemmli buffer; they were boiled for 8 min and subjected to 10% SDS-PAGE, and were then electroblotted on a nitrocellulose membrane (BioRad, Richmond, CA). The membrane was blocked for one hour at room temperature with PBS that contained 5% dry non-fat milk powder (Bio-Rad, Richmond, CA) and 0.1% Tween 20 (Merck, Darmstadt, Germany). 

Western blot was performed using mouse anti-E-cadherin (Abcam, Cambridge, UK) and anti-Integrin α6β4 (Creative Biolabs, Shirley, NY, USA) monoclonal antibodies at a dilution of 1: 250 in PBS buffer containing 5% non-fat dry milk powder and 0.1% Tween 20 for 60 min at room temperature. Incubation with an HRP-conjugated goat anti-mouse secondary antibody (Santa Cruz Biotechnology, Santa Cruz, CA, USA) at a dilution of 1:10,000 was performed under the same conditions. SuperSignal West Pico Chemiluminescent (ThermoFisher Scientific, Waltham, MA, USA) substrate was used to detect immune complexes. Mouse monoclonal anti-β-actin antibody (Merck, Darmstadt, Germany) was used at a dilution of 2500 to normalize the Western blot analysis. The bands obtained with the Western blot were subjected to densitometric analysis (BioRad, Richmond, CA, USA) and quantified. The average values for the individual samples were normalized based on the values of the actin signal to compensate for any variation in the load and concentration of the protein.

### 2.7. Statistical Analysis

Data analysis was performed using GraphPad Prism software version 8.00 for Windows (GraphPad Software, versione 8.00, San Diego, USA). Differences between groups based on cell viability assays were established using Mann–Whitney test or two-way analysis of variance (ANOVA). ANOVA with *p*-value correction was used for comparison of more than 2 groups, while comparisons between 2 groups were analyzed by the Mann–Whitney test. A *p*-value < 0.05 was considered statistically significant.

## 3. Results

### 3.1. SEM and EDS Analysis

**Figure 1:**
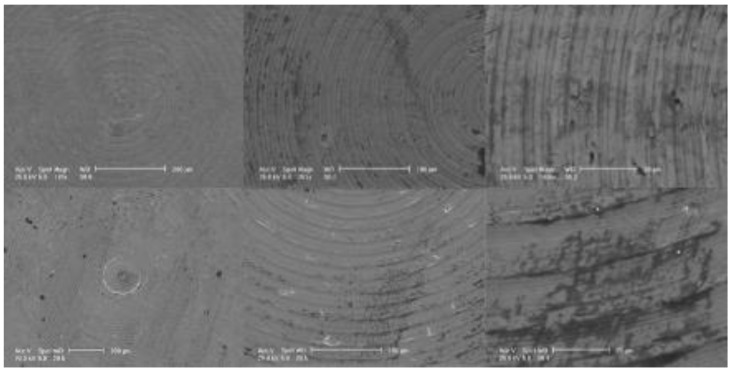
SEM analysis. The images of T1 (top) and T2 (bottom) were acquired at different level of magnification: 150×, 1000×, and 3000×.

We found that even if substantial surface differences were not appreciable at lower magnifications, at high magnification, it was evident that T2 was smoother and more homogeneous than T1 (Figure 1). This observation may probably be explained due to PVD coating with zirconium. The PVD coating homogenized and covered the residual roughness of the machined surface of T1.

**Figure 2:**
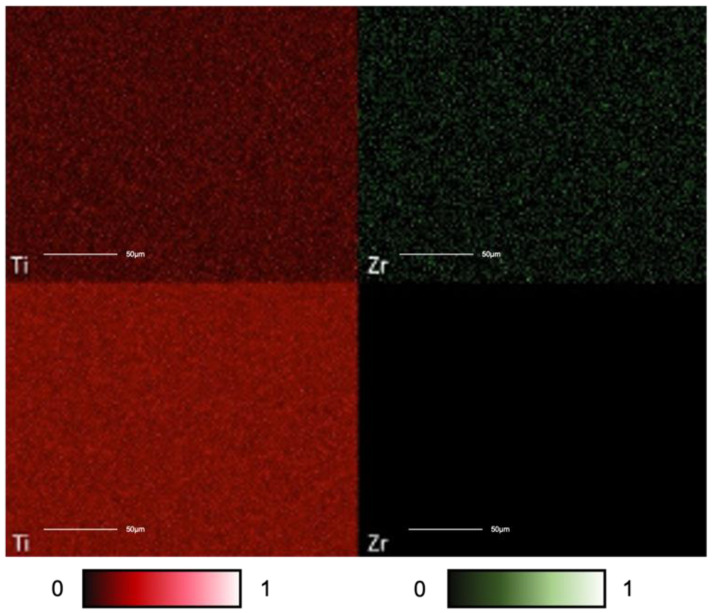
EDS elemental maps. The analysis was performed to evaluate the presence of both titanium (Ti) and zirconium (Zr) on the surface of the discs. The pictures on the top show the map distribution of the elements on the coated samples (T2), while the ones on the bottom are for the uncoated samples (T1). Each element is presented with different color intensity according to its distribution within a greyscale. Titanium (Ti) and zirconium (Zr) are presented in red and in green, respectively. As expected, zirconium (Zr) was absent on T1 surfaces. Scale bar 50μm.

The EDS images showed the presence of titanium in both samples, even if to a greater extent on T1 (Figure 2). Zirconium, as expected, was found only on T2 surfaces. The analysis shows that the coating’s surface distribution was homogeneous and uniform over the entire area measured.

### 3.2. Cellular Vitality

**Figure 3:**
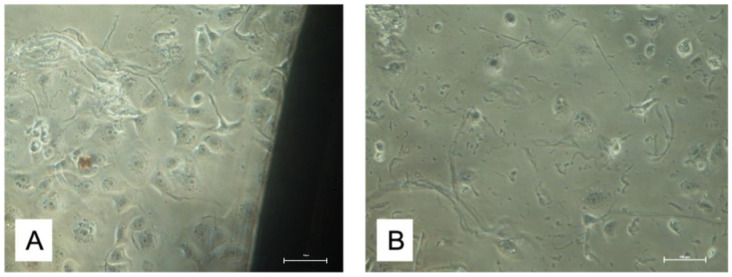
HUKE cell cultures. The imagines were obtained from T1 (**A**) and T2 (**B**) cultures at 24 h.

HUKE cells were incubated with discs at 37 °C for three durations: 24, 48, and 72 h (Figure 3).

**Figure 4:**
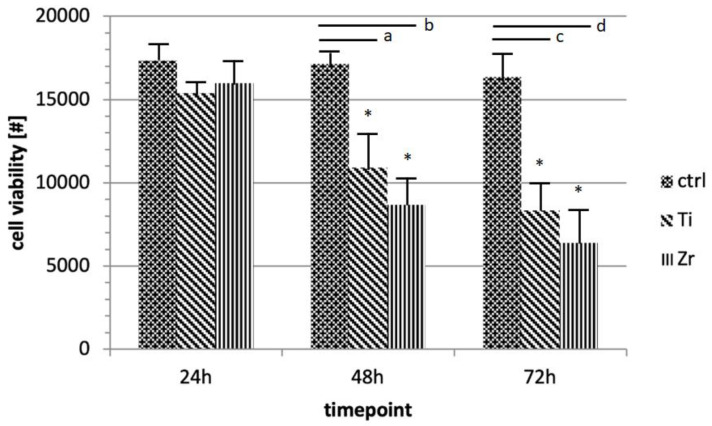
Trypan blue assay. The graph represents cell viability of the specimens (T1, T2, and C) at different time points (24, 48, and 72 h). Values are expressed as mean ± standard deviation (* *p* < 0.05): (a) Ti versus control, 48 h, *p* < 0.018; (b) Zr versus control, 48 h, *p* < 0.002; (c), Ti versus control, 72 h, *p* < 0.005; (d) Zr versus ctrl, 72 h, *p* < 0.001.

**Figure 5:**
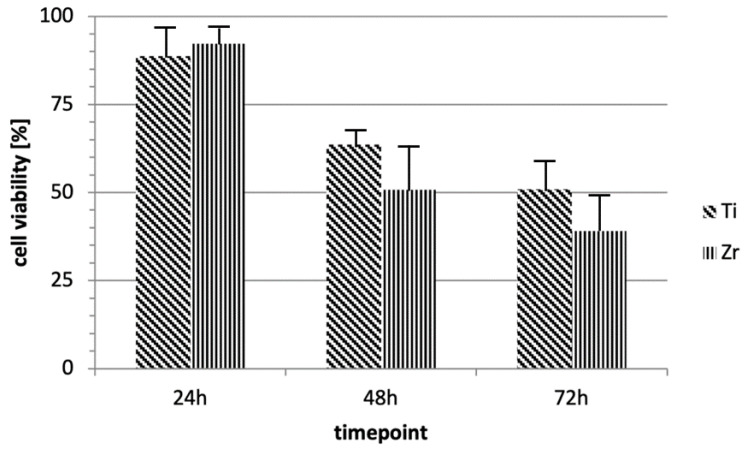
MTT assay. The graph illustrates cell viability of T1 or T2 compared with that of C at different time points (24, 48, and 72 h) of incubation with discs. Each bar represents percentage (mean ± standard deviation) with respect to the control (C).

Using trypan blue assay, cell viability was evaluated for each sample (C, T1, and T2) at different time points: 24, 48, and 72 h. Analogously, MTT evaluation was performed after 24, 48, and 72 h of incubation of HUKE cells with discs. The results revealed that cells in contact with the two materials (T1 and T2) were significantly (*p* < 0.05) less viable compared with those in contact with C, but we did not find any difference between the test samples (Figure 4 and Figure 5). This indicated that even if both materials significantly and negatively affect biocompatibility due to their ability to hinder the normal cell vitality of keratinocytes, there was no substantial differential effect between T1 and T2.

### 3.3. DAPI

**Figure 6:**
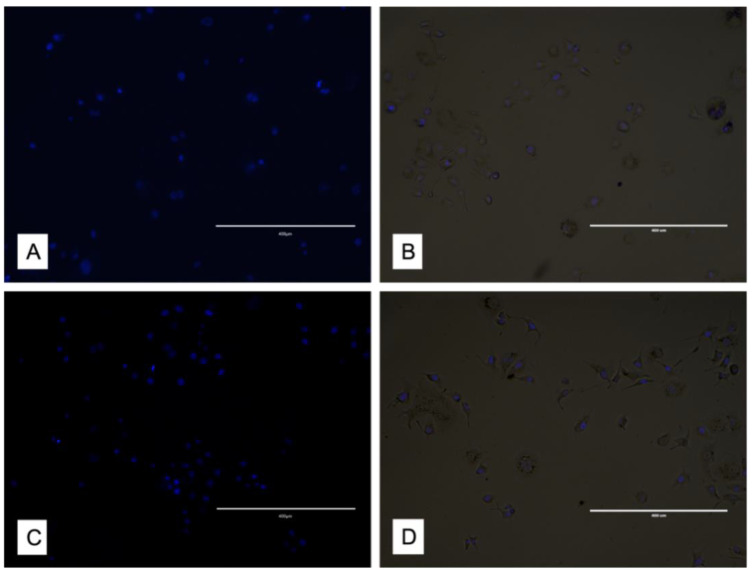
DAPI analysis highlights the nuclei of cells from the samples: (**A**,**B**) culture in presence of zirconium at 24 h; (**C**,**D**) culture in presence of titanium at 24 h. The figures on the left (**A**–**C**) were obtained through blue light, while those on the left (**B**–**D**) were through white light. Scale bar 400 μm.

Cell cultures were analyzed with DAPI staining (Figure 6). The results confirmed that cell nuclei were not influenced/affected by contact with discs. Indeed, they displayed the same morphology and aspect.

### 3.4. Western Blot

**Figure 7:**
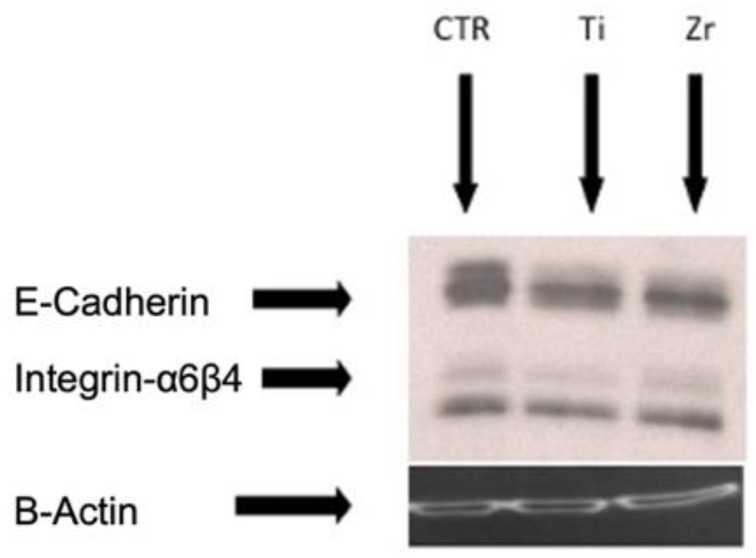
Western blot showing E-cadherin and Integrin-α6β4 expression from cell lysate of keratinocyte cultures. The adhesion molecules were expressed in a similar manner in C, T1, and T2.

Western blot was performed on proteins extracted from the cell lysate of keratinocyte cultures. Analysis showed that the adhesion molecules E-cadherin and Integrin-α6β4 were equally expressed in untreated keratinocytes, T1, and T2 (Figure 7). This indicated that both T1 and T2 with zirconium promote cell adhesion of keratinocytes without significant differences compared to the monolayer of cells alone used as reference control.

## 4. Discussion

In the present study, uncoated machined titanium Ti-6Al-4V grade 5 and zirconium-coated titanium Ti-6Al-4V grade 5 discs were compared. The first represents the most-frequently used material for dental implant abutments [40]. Zirconium is widely used in dental prothesis due to its biomechanical, esthetic, and biological properties [41,42]. Recently, zirconium coating of titanium abutments has been introduced due to its biocompatibility and antibacterial activity [43,44].

In this study, SEM showed that at lower magnification (150×), there were no differences between the two surfaces. Nevertheless, at higher magnification (3000×) it was possible to observe that the zirconium-coated surface was smoother and more homogeneous than the uncoated one. During PVD, the zirconium particles probably covered the micro-roughness of the titanium surface. EDS evaluated the functional groups on the two surfaces and showed that the zirconium coating was uniformly distributed on the titanium surface, confirming what had already been observed via SEM.

The results obtained in this study appear to be in line with the literature. A study by Größner-Schreiber et al. [45] found that ZrN-coated titanium surfaces were less rough and were smoother than uncoated ones. Brunello et al. [46] also reported that surface roughness was higher in c.p. titanium compared to that coated in ZrN but without a statistically significant difference.

Epithelial cells proliferate better along smooth surfaces. This means that the zirconium coating, by making the titanium surface smoother, should improve the adhesion of epithelial cells to the abutments. However, most articles in the literature on zirconium-coated titanium surfaces concern osteoblasts [47,48,49,50,51] and fibroblasts [45,46,52,53]. Therefore, there is a lack of in vitro studies on the behavior of epithelial cells.

In this study, the viability of epithelial cell cultures obtained from the samples was analyzed at different time points. Trypan blue and MTT analyses did not show statistically significant differences between the cultures from T1 and T2. Finally, Western blot showed that the cells in contact with T1 and T2 expressed both E-cadherin and Integrin-α6β4 in a similar way.

The present study has some limitations, in particular related to the reduced simple size. Moreover, experimental evaluation associated with cellular assays, such as MTT, did not distinguished between cells grown in contact with the disc surface and those on the well plate bottom, thus increasing the background noise. However, the results of our study confirm that zirconium-coated titanium surfaces provide a similar substrate to that of uncoated surfaces for keratinocytes adhesion and proliferation, even if significant signs of inhibition of cell viability related to both materials were reported. Concerning this last point, it would be interesting to explore the cause of this cellular effect through analysis of molecular pathways underlying apoptosis and cell-cycle regulation in a future study.

## 5. Conclusions

Recently, zirconium coating of titanium abutments has been introduced to improve the quality of the transmucosal path of dental implants. The present study showed that epithelial cells can adhere and proliferate to uncoated and PVD ZrN-coated titanium surfaces without statistical difference. Furthermore, Western blot indicated that the E-cadherin and Integrin-α6β4 expressions were similar in both specimens. There was no sign of epithelial cell toxicity to the zirconium-coated surface, proving its safety. However, most in vitro studies in the literature concern the response of gingival fibroblasts and osteoblasts to PVD zirconium-coated titanium surfaces. In the future, further in vitro studies using epithelial cells will be required to confirm our findings.

## Data Availability

The data presented in this study are available on request from the corresponding author.
